# Spekulative Forensik. Verdacht und erzählerische Imagination in Edmond Locards *Die Kriminaluntersuchung und ihre wissenschaftlichen Methoden*

**DOI:** 10.1002/bewi.70003

**Published:** 2025-10-13

**Authors:** Arne Sander

**Affiliations:** ^1^ Remarque Institute New York University New York NY 10011 USA

**Keywords:** forensic science, detective fiction, hermeneutics of suspicion, speculative science, abduction, Poe, Locard

## Abstract

Edmond Locard's *L'enquête criminelle et les méthodes scientifiques* marks a pivotal moment in criminology's transformation from a largely unmethodical practice to a scientific discipline. While Locard is best known for advancing laboratory methods of forensic analysis, this article argues that at the heart of his conception of forensics lies the assertion that it is not rationality, but vivid imagination that makes or breaks the criminal investigation. Following Locard's claim that one of the most crucial challenges in teaching forensics is to introduce fellow criminologists to the art of using intuition and creativity for problem‐solving, this article examines the concrete ways in which *L'enquête criminelle* attempts to actively engage the reader's imaginative faculty by presenting problems that can only be solved through “lateral thinking” and “abductive reasoning.” To introduce his speculative methods, I argue, Locard borrows from detective fiction in two ways: Firstly, he counterfactually presents literary case studies by Poe as real‐world cases, endorsing Dupin's detective technique as a viable criminological practice. By planting hidden clues and red herrings in semiotic puzzles to be deciphered by the reader, secondly, Locard appropriates narrative techniques to sharpen his reader's hermeneutics instincts.

## I.

*„Die Klugheit vermeidet Fehler; die Einbildungskraft aber führt zum Erfolg.*“^1^

Edmond Locard

Als der französische Kriminologe Edmond Locard auf dem spärlich eingerichteten Dachboden eines Lyoner Polizeipräsidiums im Juni 1910 die Fingerabdrücke der Tatverdächtigen Fabry und Rollin auf einer Blumenvase wiedererkennt, die er am Tatort eines Raubüberfalls in der Rue Ravat sicherstellen konnte, deutet sich eine Zäsur in der Geschichte der modernen Kriminalwissenschaft an. Denn die Übereinstimmung der feinen Poren, der Fingerlinien und der winzigen Narben eines vielfach vergrößertem Rußabdrucks ihrer Daumen mit den Konturen jener Finger, die sich auf der gläsernen Oberfläche der Vase abzeichnen, ist nicht nur der Schlüssel zum Rätsel, wer den verhältnismäßig belanglosen Diebstahl an der Lyoner Witwe André begangen hat, sondern auch der erste nachweisliche Triumph eines besonderen Beobachtungsverfahrens, einer kriminalwissenschaftlichen Methode, und eines epistemologischen Modells, deren Ermittlungserfolge bis dahin nur im Reich der von Locard verehrten Literatur Edgar Allan Poes dokumentiert waren.^2^

Der Einfluss, den Locards Fund auf die europäische Rechtsprechung des 20. Jahrhunderts hat, ist kaum zu überschätzen. Das Verfahren gegen Fabry und Rollin führt, so rühmt sich Locard, zur ersten Verurteilung an einem französischen Gerichtshof, die nicht aufgrund von Zeug:innenaussagen, Geständnissen oder sogenannten „unwiderlegbaren Vermutungen“,^3^ sondern allein auf der Basis eines physikalischen Beweismittels gefällt wird.^4^ Locards provisorisch eingerichteter Dachboden, bald schon als weltweit erstes amtliches Labor zur wissenschaftlichen Untersuchung von Beweismaterialien anerkannt, wird zu einem der institutionellen Geburtsorte der modernen Forensik – und Locards Name wird zur Chiffre für das Methodenprogramm dieser neuen Wissenschaft des Verdachts, die die Strafverfolgung auf die Entzifferung kaum sichtbarer Spuren, versteckter Indizien und scheinbar nebensächlicher Zeichen verpflichtet.^5^

Im folgenden Aufsatz soll Edmond Locards Abhandlung *Die Kriminaluntersuchung und ihre wissenschaftlichen Methoden* (*L'enquête criminelle et les méthodes scientifiques*) untersucht werden. Als eine der ersten Einführungen in die „wissenschaftlichen Grundlagen“^6^ der modernen Forensik bietet Locards *Kriminaluntersuchung* einen aufschlussreichen Einblick in jene Phase, in der sich die Kriminalistik allmählich von einer theoretisch ungebundenen Praktik und einem Erfahrungswissen zu einer modernen wissenschaftlichen Disziplin entwickelt. Obwohl diese Disziplin mit dem Versprechen antritt, gerade durch den Einsatz moderner Technologien und wissenschaftlicher Methodik im Stande zu sein, der chronischen Unzuverlässigkeit des Inquisitionsverfahrens eine objektivere Größe entgegenzustellen, setzt sich mit der modernen Forensik nach Locard ein Methodenprogramm durch, so meine These, das weder gänzlich objektiv noch wissenschaftlich genannt werden kann, sondern darauf ausgerichtet ist, empirische „Tatsachenbeweise“ spekulativ zu deuten – und fehlende Informationen, empirische Lücken und Widersprüche innerhalb des auszuwertenden Beweismaterials durch eigene Mutmaßungen zu ergänzen.^7^ Eben an der Stelle, an der ein geschickter Einsatz von Intuition, Empathie und Einbildungskraft eine nüchterne Faktenanalyse ablöst, schreibt sich mit Locards Methoden eine Kritik des Offensichtlichen in die moderne Geschichte der Detektion ein: Im Gegensatz zu jenem evidenzbasierten Modell, das in der englischen Wendung „seeing is believing“ zum Ausdruck kommt, wird die Wahrheit in Locards Modell zwingend hinter dem trügerischen Schein der Oberfläche vermutet; dort also, wo zunächst fast nichts zu sehen ist, wo der Oberflächeneindruck täuscht und Bedeutung erst aus der Latenz hervortreten muss. Um letzteres zu bewerkstelligen, müssen Aussagen über die vermeintliche Wahrheit eines beobachteten Geschehens Locard zufolge *erstens* stets unter der Voraussetzung getroffen werden, dass nichts zufällig passiert, das nichts ist wie es scheint, und das kein Zeichen bedeutungslos genannt werden kann. Und gerade dieser Modus der Interpretation macht die kriminalistische Deutung *zweitens* zur Projektionsfläche für eigene Imaginationen. Anhand von Locards Fallbeispielen soll daher im Folgenden nachvollzogen werden, wie unmittelbar die pedantische Deutung verdächtiger Zeichen, die obsessive Suche nach Korrelation, und jenes Verfahren, das Locard als „Vermutung ohne vorherige Begründung“ bezeichnet, in eine spekulative Forensik übergehen kann, in der faktische Lücken, Ungewissheiten und Widersprüche intuitiv durch Mutmaßungen und Imaginationen ergänzt und kompensiert werden.

Sich Locards Werk in diesem Sinne über seine spekulativen und pseudowissenschaftlichen Merkmale zu erschließen, sei gut begründet; hat Locards *Kriminaluntersuchung* doch wie wenige andere dazu beigetragen, den modernen Kriminalwissenschaften einen wissenschaftlichen Anspruch abzuverlangen. Ausgehend von der als Locard'sche Regel bekannt gewordenen Prämisse, dass kein Kontakt zwischen zwei Substanzen vollzogen werden kann, ohne wechselseitige Spuren zu hinterlassen, stellt Locard hier ein System der Tatortanalyse vor, das nicht nur eine neue Objektivität der Kriminaluntersuchung verspricht, sondern auch den Ausschluss des anthropologischen Faktors, jenes Anteils menschlichen Versagens also, der die Strafverfolgung bisweilen auf die Irrwege subjektiver Einbildungen und unsachlicher Interpretationsbemühungen führte. Das Potenzial moderner wissenschaftlicher Methoden der Kriminalanalyse erlaube es, so argumentiert Locard, endlich die Irrtümer und analytischen Unschärfen aus der Strafverfolgung zu verbannen, die die Befragung von Zeug:innen und Tatverdächtigen zwingend mit sich gebracht habe: zerstreute Sinneseindrücke und lückenhafte Erinnerungen; eine durch Aufregung, Ressentiments, persönliche Interessen oder Eitelkeit beeinträchtigte Wahrnehmung des Tatgeschehens; sowie bewusste Fehldarstellungen, hysterische Lügen, ein „verdorbenes Gedächtnis“^8^ und eine „Fabelsucht“,^9^ die Locard – und dies ist bezeichnend – „allen Kindern, fast allen Frauen, fast allen Nervösen“ unterstellt.^10^ Das verheißungsvolle Gegenmittel, das Locard diesem „Gift der Zeugenaussage“ („Le poison des témoignages“)^11^ entgegenstellt, ist die kriminalwissenschaftliche Zuwendung zu den „stummen Zeugen, die nicht lügen und nicht täuschen: Fingerabdrücke, Fußspuren, Nagel‐ und Zahneindrücke, verkohlte Papiere, die noch lesbar sind, Formeindrücke von Einbruchswerkzeugen, Teile von Kleidung und Haaren.“^12^

Um diese zu analysieren, greift Locard auf ein umfassendes System der Tatbestandsdiagnostik zurück, das von den analytischen Verfahren der Daktyloskopie^13^ (Fingerabdruckverfahren), der Trassologie (Spurenanalyse) und Schriftmessung, über den Einsatz labortechnischer Instrumente und chemischer Verfahren zur Untersuchung und Reproduktion sachlicher Indizien (Photographie, Lichtmikroskopie, forensische Chemie) bis zur Entwicklung bürokratischer Systeme zur Sicherung, Registratur und Klassifizierung von Tatbestandsmerkmalen, Asservaten und biometrischen Personenmerkmalen reicht.^14^

Wie im Folgenden gezeigt werden soll, umfasst Locards Methodenwerk allerdings eben nicht nur jene wissenschaftlichen Methoden, technischen Verfahren und bürokratischen Prozesse, die bis heute mit seinem Namen in Verbindung gebracht werden. Wie zu sehen sein wird, liegt im Kern der *Kriminaluntersuchung* nach Locard vielmehr der Versuch der Formalisierung von – und der praktischen Einführung in – eine Reihe von detektivischen Geschicken, deren analytisches Potenzial sich in erster Linie an dem erfolgreichen Einsatz von Intuition, Empathie und Einbildungskraft bemisst. Weil die „Beobachtung der Spuren, selbst wenn sie mit allen Hilfsmitteln der Technik durchgeführt worden ist, ein totgeborenes Kind [bleibe], wenn sie nicht durch die Vermutung belebt wird, welche die Tatumstände ordnet und erklärt“,^15^ brauche es nach Locard nämlich neben wissenschaftlichen Methoden zur Erfassung und Prüfung von Indizien in erster Linie folgende Kernkompetenzen, die sich mit Carlo Ginzburg als programmatische Merkmale des „Indizienparadigmas“ der modernen Humanwissenschaften charakterisieren ließen:^16^ ein instinktives Gespür für nebensächliche Details, in denen sich ein heimlicher Sinn zu erkennen gibt; die immersive Fähigkeit, sich in die Psyche von Täter:innen hineinzuversetzen und sich das Tatgeschehen aus deren Perspektive zu vergegenwärtigen; eine Empfänglichkeit für plötzliche Eingebungen, unwillkürliche Gedankenblitze und „unbegründete Vermutungen“; und schließlich das kombinatorische Geschick sämtliche Indizien und Mutmaßungen so zueinander in Relation zu setzen, dass im Geiste der Ermittelnden das Tatgeschehen und das Täter:innenprofil so deutlich hervortritt, als sei es direkt beobachtet worden. Um dies gewährleisten zu können, verknüpfen Locards Methoden den Anspruch auf eine *ungerichtete* Aufmerksamkeit des Beobachters – eine besondere Form der Vigilanz, in der die Aufmerksamkeit nicht auf ein bestimmtes Objekt, sondern auf ein lückenloses Erfassen der Gesamtheit scheinbar unwesentlicher Details am Rande des Geschehens gerichtet wird – mit einer Form der Interpretation, in der es nicht darum geht, möglichst sichere Schlüsse zu ziehen, sondern darum, möglichst kreative bzw. erfindungsreiche Vermutungen über Täter:in und Tatgeschehen auszusprechen. Nicht an der nüchternen Vernunft, so heißt es in der *Kriminaluntersuchung*, sondern „an der Kühnheit der Vermutung erkennt man den trefflichen Kommissar.“^17^

Eben jenen Verfahren, die Locards Handbuch der Kriminaluntersuchung vor die Aufgabe stellen, Intuition und analytische Raffinesse praktisch zu vermitteln, widmet Locard das letzte Kapitel seines Buches, das mit dem Titel „Die wissenschaftlichen Grundlagen des Tatsachenbeweises“ überschrieben ist. Während die technischen Methoden der *Kriminaluntersuchung* gut dokumentiert sind und als Beitrag zur Theoretisierung der Kriminalwissenschaften hinreichend erforscht wurden, ist dieser Teil der *Kriminaluntersuchung* bisher kaum von der Forschung untersucht worden. Gerade hier bietet Locards Werk indes nicht nur einen wertvollen Einblick in die spezifischen Praktiken der modernen Kriminalistik, sondern auch in die epistemologischen und wahrnehmungsästhetischen Prämissen, die den Anfängen dieser jungen Wissenschaft zugrunde liegen – sowie den literarischen Werken, die ihre Verfahren inspiriert haben.^18^

Mag es zunächst kontraintuitiv erscheinen, ein polizeiliches Handbuch nun aus literaturwissenschaftlicher Perspektive zu untersuchen, so ist dieses Unterfangen vielleicht damit zu rechtfertigen, dass Locards Methodenprogramm aufs Engste mit Ökonomien modernen Erzählens verbunden ist. Über ein Dutzend Mal treten in Locards Analysen Referenzen auf diverse literarische Werke auf, die von Klassikern der „Sattelzeit der Moderne“^19^ bis zum modernen Detektivroman reichen. Eine zentrale Stelle in Locards Werk nehmen dabei insbesondere die Kriminalerzählungen Edgar Allan Poes und Arthur Conan‐Doyles ein. Mag eine Referenz auf Figuren wie Poes C. Auguste Dupin und Sherlock Holmes im Kontext detektivischer Künste zunächst wenig erstaunlich erscheinen, so ist sie deshalb bemerkenswert, weil Locard nicht nur ausdrücklich die Anwendbarkeit von deren Methoden auf reale Fälle betont, sondern fiktive Fallgeschichten konsequent im selben Referenzrahmen wie reale Fallbeispiele anführt und mitunter sogar deren Fiktionalität abstreitet, wodurch ein elementarer Unterschied zwischen Faktizität und Fiktionalität nivelliert wird.

Ungleich wichtiger als diese intertextuellen Referenzen ist die Tatsache, dass Locards Fallbeispiele deutliche Anleihen an die Darstellungsverfahren moderner Detektivgeschichten nehmen. Die narrativen Muster und generischen Konventionen der Detektivgeschichte, so möchte ich darlegen, präfigurieren die Form, die Locard wählt, um das Potenzial der wissenschaftlichen Methode in der Kriminaluntersuchung darzustellen: Was Locards Fallstudien auszeichnet ist eine narrative Logik des Suspense, dessen Erfolg sich am Vorenthalt von Information, an der Latenz zwischen einem Rätsel und seiner Lösung und einer gekonnten Lenkung von Leseerwartungen bemisst.^20^ Genauer betrachtet ließe sich das narrative Schema, das Locard anwendet, auf folgende Faktoren zuspitzen: 1.) die Exposition eines Geheimnisses, dessen Aufklärung seinen Leser:innen zunächst verwehrt bleibt, um diese zur Entwicklung eigener Hypothesen zu motivieren; 2.) die identifikationsstiftende Darstellung eines detektivischen Blicks, der jedes noch so unscheinbare Detail fokussiert und die Aufmerksamkeit gekonnt auf Randschauplätze lenkt. Damit wird 3.) eine Rezeptionshaltung konsolidiert (und belohnt), die naheliegende Erklärungen weitestgehend missachtet und die eigentliche Bedeutung des Geschehens zwingend hinter der täuschenden Oberfläche des sinnlich Wahrnehmbaren vermutet. Erscheint die verräterische Wirklichkeit, die sich am Tatort abzeichnet, schließlich gänzlich von interpretierbaren Indizien durchdrungen, die vermeintlich einen heimlichen Sinn indizieren, so bemisst sich die erfolgreiche Entschlüsselung verdächtiger Zeichen schließlich 4.) an der von Lesenden durch die Lektüre einzuübenden Begabung, scheinbar disparate Zeichen, Zustände, Ereignisse und Beobachtungen intuitiv zu einer Hypothese verdichten zu können, die dem unübersichtlichen Tatgeschehen Kontur verleiht – und zwar in Form einer Geschichte.

Doch auch abseits der spezifischen generischen Gemeinsamkeiten zwischen den Darstellungsverfahren von Kriminalliteratur und den Fallbeispielen Edmond Locards, die im Folgenden noch ausführlich untersucht werden sollen, zeichnet sich in Locards wissenschaftlichen Schriften eine zweite, noch grundsätzlichere Überschneidung von Fiktion und moderner Forensik ab: Wie eingangs erwähnt, steht innerhalb der konkreten Methoden, in die Locards *Kriminaluntersuchung und ihre wissenschaftlichen Methoden* einführt, an zentraler Stelle immer wieder die Produktivkraft erzählerischer Imagination. Denn obwohl es das erklärte Ziel dieses Standardwerks der modernen Forensik ist, der „Fabelsucht“ der Zeug:innen entgegenzuwirken, die gefährliche Überzeugungskraft falscher/verzerrter Nacherzählungen des Tatgeschehens einzudämmen und unzuverlässige Augenzeugenberichte durch nüchterne wissenschaftliche Analysen zu ersetzen, beruhen Locards Verfahren der Beweisführung entscheidend auf Prozessen, die das Fabulieren aktiv in den Prozess der Beweisführung einbinden und die Vorstellungskraft zum zentralen Baustein der Kriminaluntersuchung erklären.

Um dies darzulegen, sollen im Folgenden zunächst zwei konkrete Verfahren untersucht werden, in die Locard seine Leser:innen einzuführen versucht: die „peinlich genaue Beobachtung“ und die „Annahme ohne vorherige Begründung“. Da Locard diese Verfahren nicht nur theoretisch erläutert, sondern seine Leser:innen anhand konkreter Fallbeispiele ganz praktisch in die Kunst des Beobachtens und Vermutens einführt, soll im abschließenden Teil gezeigt werden, wie er gezielt ihre Vorstellungskraft herausfordert, indem er ihnen Rätsel stellt, die nur durch laterales Denken und abduktives Schließen zu lösen sind.

## II.

Locards Einführung in die „wissenschaftlichen Grundlagen des Tatsachenbeweises“ beginnt mit dem Versuch, seine Leser:innen von der Unerlässlichkeit einer „peinlich genauen Beobachtung“ der Tatsachen zu überzeugen. Beim „Beobachten“ handelt es sich um die erste von vier Methoden, die Locard in seinem polizeilichen Handbuch als neue „wissenschaftliche Verfahrensarten“^21^ der Kriminaluntersuchung vorstellt. Was aber heißt *beobachten* für Locard? Und worin bestehen die Unterschiede dieses spezifischen Beobachtungsverfahrens zu den konventionellen, bis dahin erprobten Verfahren der Spurensicherung?

Zunächst einmal gibt Locard vor, dass es sich bei seinem Begriff des Beobachtens nicht primär um eine Fähigkeit handelt, sondern um ein kalkuliertes Vorgehen. „Beobachten“, so definiert er, „das bedeutet nicht ein Ansehen auf gut Glück, bedeutet nicht nur Kenntnis nehmen, was den Blick auf sich zieht, worauf der Blick ruht; es ist ein planmäßiges Nachspüren nach vorher festgelegtem Plan.“^22^ Ferner scheint sich der Wert dieses planmäßigen Nachspürens weniger an dem qualitativen Anspruch zu bemessen, diejenigen Spuren zu verzeichnen, die für die Rekapitulation des Tatgeschehens am ergiebigsten erscheinen, als an der quantitativen Prämisse, möglichst lückenlos *alles* zu verzeichnen, was am Tatort zu sehen ist. Weil, wie Locard etwas tautologisch formuliert, „die sorgfältigste Untersuchung der Spuren, mögen sie auch noch so winzig sein, […] für die Beweisart das Grundgebot ist“,^23^ verlange es „ein Suchen und Beschreiben bis in die Einzelheiten hinein alles dessen, was zur Lösung des verbrecherischen Rätsels beitragen könnte“:^24^

Von der Beobachtung muss man […] verlangen, dass sie genau und peinlich ist. […] Die Beobachtung ist genau, wenn jede Feststellung mit aller Sorgfalt und bis in die Kleinigkeiten hinein getroffen wurde […]. Die Beobachtung ist peinlich genauer, wenn kein Umstand vernachlässigt worden ist.^25^

Es ist nicht weniger als ein Grundgebot dieses pedantischen Beobachtungsverfahrens, dass die Gesamtheit dessen, was mit dem Blick erfasst werden kann, potenziell signifikant sein könnte. Jedes Detail, sei es auch noch so unscheinbar, verlangt die Aufmerksamkeit des Ermittelnden und kein Zeichen kann von vornherein bedeutungslos genannt werden. Gerade weil hinter jedem Zeichen eine verbrecherische Absicht, ein fahrlässiger Fehler oder eine verborgene Intention seines Urhebers vermutet werden muss, sollte in diesem ersten Schritt der Kriminaluntersuchung jedwede Form von Koinzidenz und Bedeutungslosigkeit vorübergehend ausgeschlossen werden – alle Zeichen sind miteinander verbunden und selbst dem Fehlen einer Spur muss ein verbrecherisches Kalkül unterstellt werden.

Die Arbeitshypothese, dass alles *bedeutet*, nichts zufällig passiert und alle Zeichen in Relation zum Tatgeschehen stehen, nimmt, wie im Folgenden zu sehen sein wird, einen entscheidenden Einfluss auf die Art und Weise, wie verdächtig(t)e Zeichen interpretiert und Vermutungen ausgesprochen werden. Es geht hier nämlich gerade nicht darum, die Signifikanz oder Irrelevanz eines jeweiligen Einzelzeichens in Relation zur Tat zu beweisen – dieser Beweis wird erst retroperspektiv mit der Lösung des Falles erbracht –, sondern es gilt, diejenige Hypothese zu finden, die der Gesamtheit des Beobachteten möglichst ganzheitlich Rechnung trägt und also eine Korrelation zwischen möglichst vielen Bedeutungsträger erkennbar macht – unabhängig davon, ob diese in einem sachlich begründbaren Zusammenhang zur Tat stehen oder nicht.

Auch wenn Locard jedwedes Detail, das in den Blick der Ermittelnden fällt, unter der Maßgabe betrachtet, potenziell den Schlüssel zu dem zu lösenden Rätsel darzustellen, was am Tatort passiert ist, schleicht sich jedoch, ohne dass Locard dies reflektiert, eine qualitative Hierarchisierung vermeintlicher Indizien in seiner Methode ein. Dabei bemisst sich die Qualität eines Zeichens für ihn jedoch keineswegs an dessen Deutlichkeit oder unmittelbaren Evidenz für das Tatgeschehen. Im Gegenteil sind es für Locard in erster Linie die unscheinbaren Details, welche die erfolgversprechendste Passage vom Mysterium zur Lösung in Aussicht stellen. „Im Allgemeinen ist ein Zeichen um so wesentlicher, je versteckter es ist“,^26^ stellt Locard in Bezug auf die Schriftstückanalyse fest. Es gibt vielleicht keinen Satz, der das Grundprinzip der forensischen Hermeneutik nach Locard auch außerhalb dieser spezifischen Anwendung derart pointiert zusammenfasst. „Zahlreiche Beispiele haben den Blick dafür geöffnet“, führt Locard weiter aus,

daß selbst die *fragwürdigsten* Spuren zur Klärung einer schwierigen Frage beitragen können. Wer weder einen Kerzenfleck noch Zigarettenasche noch einen Kratzer auf irgendeiner Politur noch ein Riss in Spinnwebfeinem Stoff vernachlässigt hat, wird nur seine Pflicht als Beobachter erfüllt haben. Er wird vielleicht die zuverlässigste Grundlage für Annahmen und Schlussfolgerungen gewonnen haben, die zur Wahrheit führen können.^27^

Kann die nicht direkt erfahrbare Wahrheit eines Tatgeschehens potenziell gerade durch eine Analyse der vermeintlich unscheinbarsten und strukturell fragwürdigsten Spuren rekonstruiert werden, so zeugen Locards Ausführungen von einem Erkenntnisprozess, den Carlo Ginzburg als „konjekturales Paradigma“ bezeichnet hat. Im Verlauf des neunzehnten Jahrhunderts tauchte, so Ginzburg, „fast unbemerkt ein epistemologisches Modell (oder, wenn man will, Paradigma) im Bereich der Sozialwissenschaften [auf]“,^28^ das „sich dadurch auszeichnet, daß [es] den Sprung von scheinbar unerheblichen Fakten, die der Beobachtung zugänglich sind, zu einer komplexen Realität ermöglicht, die ihrerseits – zumindest direkt – nicht sichtbar ist.“^29^

Anders als das galiläische Prinzip, welches auf der Wiederholbarkeit des Einzelfalles und auf der Formulierung allgemeingültiger Prinzipen gründet, legitimiert sich das konjekturale Paradigma im Hinblick auf all jene Erfahrungsbereiche, in denen Realität weder direkt erfahrbar noch generalisierbar ist und experimentelle Versuche nicht reproduziert werden können. Ob medizinische Diagnostik, Psychoanalyse, Kriminologie oder Stilkritik – in den konjekturalen Wissenschaften wird versucht, Wahrheit durch die geschickte Deutung von Einzelfällen zu finden, die ihrerseits lediglich durch die Analyse von scheinbar nebensächlichen Spuren, Symptomen und Hinweisen rekonstruiert werden können.

Charakteristisch für das konjekturale Modell ist dabei nicht nur der Schluss von einer Wirkung auf deren Ursache, sondern auch die zentrale Rolle der Vorstellungskraft. Die imaginative Fähigkeit, intuitiv unbewegte Zeichen in die Sukzession einer Bewegung zu setzen und sich einen Handlungsverlauf vorzustellen, der die Verursachung der Spuren retrospektiv erklären würde, wird zur Voraussetzung der Identifikation einer Ursache. Das Deuten von Zeichen konvergiert hier immer bereits mit der kulturellen Praxis des Erzählens, wie Ginzburg am Beispiel der prähistorischen Kunst des Fährtenlesens darlegt, in der er einen möglichen Ursprung des Indizienparadigmas vermutet:^30^ „Der Jäger war somit vielleicht der erste, der ‚eine Geschichte erzählte‘“, so Ginzburg, „da es ihm gegeben war, aus den stummen (kaum wahrnehmbaren) Zeichen, die seine Beute hinterließ, eine kohärente Ereignisfolge herauszulesen.“^31^

Um eine versteckte Wahrheit, eine Gestalt oder eine Geschichte aus einer Spur rekonstruieren zu können, braucht es also neben dem pedantischen Eifer jedwedes Zeichen als potenzielles Indiz zu verstehen gleich zwei Fähigkeiten, die weder generalisierbar noch vollständig systematisierbar sind: erstens die Gabe innerhalb eines kulturell determinierten Zeichensystems intuitiv diejenigen nebensächlichen Details ausfindig zu machen, aus denen sich Fakten über die Gestalt des gesuchten Objekts oder des Tatbestands ableiten lassen; zweitens eine gewisse poietische Begabung oder Vorstellungskraft, die es ermöglicht, die scheinbar unerheblichen Fakten so „zu ordnen, daß sie eine Erzählfolge bilde[n]“.^32^

Locards *Kriminaluntersuchung* entspricht dieser Konzeption mustergültig. Der ideale Ermittler nach Locard zeichnet sich durch die Versessenheit aus, unnachgiebig nach Zeichen einer verborgenen Wahrheit zu suchen. Vor allem aber verbindet er die Fähigkeit zum intuitiven Erkennen der Wahrheit im Nebensächlichen mit der, so Locard Edgar Allen Poe zitierend, „Einbildungskraft des Dichters“.^33^ Die „vollkommene Beherrschung der Technik“^34^ des Beobachtens besteht für Locard nämlich bemerkenswerterweise nicht allein darin, möglichst akribisch Sachverhalte zu registrieren oder möglichst nüchtern Schlüsse aus diesen Beobachtungen zu ziehen, sondern sie bemisst sich nach dem imaginativen Vermögen der Ermittelnden gewagte Spekulationen anzustellen: „Die Einbildungskraft, die sogar bei mathematischen Verfahren eine Rolle spielt, kann auch bei der Kriminaluntersuchung nicht ausgeschlossen werden. Ganz im Gegenteil, an der Kühnheit der Vermutungen erkennt man den vortrefflichen Polizeibeamten.“^35^

Die Empfänglichkeit für plötzliche Eingebungen sei dabei laut Locard weder durch Disziplin noch durch Regeltreue zu erlernen. Im Gegenteil setze die spekulative Raffinesse eine gewisse Bereitschaft zum Bruch mit den tradierten Normen und der Lehrmeinung voraus.

Denn die beste Art, etwas zu finden, besteht darin, dass man zu suchen versteht; dazu gehört vor allem ein gewisses Maß von eigenem Anschauungsvermögen oder wenn man will von Begabung, das dem Verstande die Möglichkeit gibt, *unter den nebensächlichen Einzelheiten gerade diejenigen herauszusuchen, die kein Lehrbuch, kein Unterricht vorausgesehen hat*, von denen aber gerade die Aufklärung ihren Ausgang nimmt. Einer Technik, die noch so jungfräulich, noch im Werden begriffen ist, gibt es wohl kaum eine Maßnahme, bei der ein gut veranlagter Kopf während der ersten Beobachtungen nicht eine erfolgbringende [sic!] Einzelheit herausfinden könnte, die einem geschulteren Sucher entgehen würde, dem eine weniger gute Veranlagung zur Seite steht.^36^

Eine gute Beobachtung ist nach Locard zuvörderst eine Einstellungssache, die sich wesentlich durch den Bruch mit normativen Prämissen und kanonisiertem Wissen („kein Lehrbuch, kein Unterricht“) auszeichnet. Peinlich genau zu beobachten bedeutet, gerade diejenigen Einzelheiten zu betrachten, vor denen der geschulte Blick versagt.

Insofern sich die Meisterschaft der Beobachtenden für Locard durch Eigenschaften wie Autonomie, Regelbruch, Originalität und Innovation auszeichnet, vereint Locards idealer Ermittler das Leistungsprofil des modernen Subjekts/homo oeconomicus, das sich an Fortschrittsorientierung, Improvisationstalent und einer Begabung zum lateralen Denken^37^ bemisst, mit dem Selbstverständnis des modernen Dichters, dessen ausgeweitete poetische Lizenzen spätestens seit dem Geniekult des 18. Jahrhunderts den Regelbruch und die Abweichung zur Norm nicht nur legitimieren, sondern regelrecht zum notwendigen Merkmal der Kunst machen.^38^ Locards Kriminalist ist ein modernes Genie. Und seine Tätigkeit/Begabung ist nicht weniger Kunst als Wissenschaft.

## III.

Nachdem man im ersten Schritt alle auffälligen Einzelheiten und nebensächlichen Details registriert hat, gilt es im zweiten Schritt der Kriminaluntersuchung eine Erklärung zu finden, die es ermöglicht, alle dieser auffälligen Einzelheiten so zueinander in Relation zu setzen, dass aus dem vorhandenen Anschauungsmaterial eine Hypothese abgeleitet werden kann, welche „die von der Inaugenscheinnahme zusammengetragenen Tatsachen in verständigen Zusammenhang bringt.“^39^ Die kriminalistische Methode, die dies leisten soll, nennt Locard das „Verfahren der Annahme ohne vorherige Begründung“.^40^ Die Bezeichnung ist nicht zuletzt deswegen treffend, weil sie die spekulative Dimension des Verfahrens impliziert. Nicht Kausalität, Evidenz oder logische Begründungen kennzeichnen den Schluss vom verdächtigen Zeichen auf seine vermeintliche Bedeutung für die Tat, sondern unmittelbare Formen der Eingebung, die jeder kritischen Reflexion vorausgehen.

Anstatt von den konkreten Spuren qua Hypothesenbildung zu den Bedingungen ihrer Verursachung voranzuschreiten, sucht Locards Methode genau umgekehrt, qua Beobachtung nach fasslichen Konkretionen für eine immer schon vorauseilende Vermutung. Denn es handelt sich bei der Annahme, wie Locard erklärt, um eine „zunächst unbegründete Vermutung, die *vor* jeder Beobachtung aufgestellt wird und durch letztere nur nachgeprüft wird.“^41^ Die Mutmaßung ist hier also nicht das Produkt der Beobachtung eines verdächtigen Indizes, d.h. die Wirkung dessen, was beobachtet wird, sondern der Ausgangpunkt einer jeden Beobachtung. Oder anders formuliert: Verdacht ist die ursächliche Prämisse, unter der Beobachtung stattfinden muss.

Die Vermutung sei in der Kriminalistik der Abschnitt, so Locard, für den man am schwersten Regeln aufstellen könne: „in dem verdienstlichen Wirken des untersuchenden Beamten bildet sie den Anteil des ‚Angeborenen‘. Im Grunde ist es das, was man *Witterungen* zu nennen pflegt.“^42^ Sei man aber mit der Gabe natürlicher Einbildungskraft ausgestattet, so Locard, könne man in gewissen Fällen sogar Rätsel lösen, ohne jemals eigene Beobachtungen angestellt zu haben. „Denn nicht im tiefen Tale kann man die Wahrheit finden“, zitiert er Poe, „sondern nur beim Ausblick von der Höhe des Berges.“^43^

Sein Paradebeispiel für den Erfolg einer rein spekulativen Kombinatorik ist der Fall Marie Rogêt, deren Leiche in Poes Kurzgeschichte *The Mystery of Marie Rogêt* in der Seine gefunden wird. Der Fall, bei dem es sich, wie Locard seinen Leser:innen versichert, keinesfalls um eine Erfindung handele, sondern um „eines der dunkelsten Rätsel, das je ein Polizeibeamter zu lösen hatte“,^44^ beruht auf einer wahren Begebenheit, die sich ein Jahr, bevor Poe die Geschichte nach Paris verlegt, in New York ereignete. Nachdem die Polizei am Flussufer des Hudson Rivers, in dem die getötete Mary Cecilia Rogers gefunden wurde, die Spuren eines Kampfes, die Schleifspur eines Körpers sowie diverse persönliche Gegenstände der jungen Frau sicherstellte, fiel ihr Verdacht auf eine Gruppe junger Männer, die schließlich der Ermordung des Mädchens beschuldigt wurden. Poe, der die Ermittlungen dieses Mordfalls in den Medien verfolgte, hielt die Beschuldigung der vermeintlichen Täter jedoch für fragwürdig und entwickelte ein alternatives Erklärungsmodell, welches ihm schließlich als Grundlage für die zweite seiner „Geschichten einer Schlussfolgerung“ (*tales of ratiocination*) diente.

Die fiktiven Anteile dieses literarischen Dokuments konsequent ausblendend, präsentiert Locard die Vermutungen, die Poes Detektiv Dupin in der diegetischen Welt anstellt, vorbehaltslos als Lösung des realen Falls. „Ich erinnere daran, dass in Wirklichkeit Dupin Edgar Poe selbst ist“,^45^ schreibt Locard. Poe habe „nach demselben System der zunächst unbegründeten Vermutungen, welches er dem Dupin unterstellte, in seinem Kämmerlein, ohne vorherige Beobachtungen und ohne andere Grundlagen als die Ausführung der Tageszeitungen, tatsächlich den realen Täter entdeckt.“^46^

Möglicherweise beruft er sich hier auf ein Zitat Poes, der in einem Brief schrieb: „under the pretense of showing how Dupin […] unravelled the mystery of Marie's assassination, I, in fact, enter into a very rigorous analysis of the real tragedy in New York“.^47^ Während aber in der Realität nie ein Täter überführt werden konnte und selbst in Poes Kurzgeschichte nur Mutmaßungen ausgesprochen werden, behauptet Locard schlichtweg, dass Poe die Ermordung erfolgreich aufgeklärt – und den wahren Mörder erfolgreich überführt habe. Dupins Mutmaßungen treten bei Locard schlussendlich nicht nur als Inspirationsquelle, sondern regelrecht als Rechtfertigungsgrundlage des sogenannten Verfahrens der „Annahme ohne vorherige Begründung“ auf.^48^ So fasst Locard Poes Geschichte einer Schlussfolgerung folgendermaßen zusammen:

Er [Dupin/Poe, A.S.] weist nach, und zwar stets aufgrund seines zergliedernden Untersuchungsverfahrens und ohne Nachprüfung durch unmittelbare Inaugenscheinnahme, daß das junge Mädchen, wenn es von der Bande angefallen worden wäre, keinen Widerstand hätte leisten können und daß es dann keine Kampfspuren gegeben hätte, daß andererseits mehrere Männer, die eine Leiche wegschaffen wollen, den Körper nicht geschleift, sondern getragen hätten, daß sie nicht schwer belastende Gegenstände am Tatort hätten liegen lassen, daß sie eine Einfriedung nicht durchbrochen, sondern den Körper darüber gehoben hätten, daß sie schließlich nicht darauf gekommen wären, um den Hals des Opfers einen Strick zu binden, um es daran zu ziehen; das deutet vielmehr auf einen einzelnen Mann hin, der von der Anstrengung erschöpft war, die der Transport eines erheblichen Gewichts erforderte. Marie war also nicht den Rohheiten einer Bande, sondern eines einzelnen Mannes zum Opfer gefallen. Wer aber war es? Er hat sofort in einem Boote, das zu dem Verbrechen benutzt worden war, verschwinden können; Er hatte also freien Zutritt zu dem Tatort und war dort Herr. Andererseits war der Mann, mit dem das junge Mädchen [fünf Monate vor dem Drama, A.S.] beim ersten Mal geflohen war, ein Seeoffizier; die Zeiten, welche die beiden Fälle des Verschwindens trennte, entsprachen der Dauer einer gewissen Kreuzerfahrt. Nun konnte also der Täter leicht festgestellt werden. Diese Vermutung war einzigartig, richtig; […]. Nach Lage der Sache muß man sich dem beugen. Das Verfahren der Annahme ohne vorherige Begründung hat seine Daseinsberechtigung erwiesen.^49^

Freilich hat diese Praxis des Schließens bereits vor ihrer Entdeckung durch Locard im Feld der Literatur längst einen Namen. Es handelt sich bei dem „Verfahren der Annahme ohne vorherige Begründung“ nämlich um nichts anderes als jene Art des Schließens, die Sherlock Holmes „deductions“^50^ nannte und im Sinne der Semiotik Charles S. Peirces vielleicht richtiger als „Abduktion“ bezeichnet werden müsste.^51^ Der abduktive Schluss kombiniert Strategien kombinatorischen Denkens mit einer „Praxis systematisierten Vermutens, die dem Raten nicht unähnlich ist“;^52^ er gesteht der äußerst unzuverlässigen kombinatorischen Verbindung überraschender Beobachtungen (d.h. Phänomenen, Ereignissen und Zeichen, die deviant zu einer a priori gefassten, normativen Voraussetzung erscheinen) ebenso viel Erkenntnispotenzial zu wie dem plötzlichen Gedankenblitz, dem intuitiven Einfall, der diese Abweichungserscheinung qua Hypothese potenziell erklärbar macht. Derart grenzt sich die Abduktion dezidiert von den syllogistischen Verfahren der Induktion und Deduktion ab:

Während in der Deduktion irgendein Gesetz oder eine Regel den Ursprung einer Ableitung darstellt und während in der Induktion dieses Gesetz als Ergebnis der Schlußfolgerung resultiert, hat es in der kriminalistischen Schlußfolgerung den Charakter einer leitenden Hypothese. Dieses Gesetz ist also weder Ursprung noch Ergebnis, sondern diejenige Annahme, mit deren Hilfe man von einem sichtbaren Sachverhalt zu einer Aussage über diesen Sachverhalt gelangt. Die Aufgabe des Detektivs besteht also nicht nur in ruheloser Beobachtung und im Aufsammeln von Indizien, sie besteht vor allem darin, schnell, geschickt und einfallsreich mögliche Gesetze zu veranschlagen, d.h. Bedeutungen zu vermuten, d.h. Verdächtigungen zu extemporieren. Es gibt in diesem Verfahren keine schlußlose Wahrnehmung, oder genauer: die Qualität einer Wahrnehmung liegt in deren Verknüpfung mit einer Regel.^53^

## IV

Fassen wir Locards Konzepte der Beobachtung und der „Annahme ohne vorherige Begründung“ zusammen, so ließe sich feststellen: Im Gegensatz zu einem konventionellen Wahrnehmungsmodell, deren Deutung von sichtbaren Ereignissen und Vorgängen in der Welt ihren Ausgang nimmt, ist das Erkennen der Wahrheit in Locards Methode der Kriminaluntersuchung nicht länger an materielle Evidenz gebunden. Bedeutung wird umgekehrt gerade dort vermutet, wo fast nicht(s) gesehen werden kann und der Schein trügt.

Locards Beobachtungsverfahren ließe sich so auf die doppeldeutige Formel „nichts als Zeichen“ zuspitzen. Denn einerseits wird die Gesamtheit des sinnlich Wahrnehmbaren zum Indiz, das auf ein verborgenes Tatgeschehen, eine verborgene Wirklichkeit und einen versteckten Sinn hin lesbar gemacht werden muss. Andererseits wird noch die Absenz eines Zeichens, das also, was fast nicht gesehen wird, was versteckt scheint, zum Zeichen einer trügerischen Wahrheit – und zum vielleicht sichersten Beweis dafür, dass etwas verschleiert wurde. In dieser Grundkonstitution des Verfahrens gibt es kein Refugium vor Bedeutsamkeit: Weil schlichtweg alles bedeutet, wird jedes Objekt der Anschauung zum Indiz und Wirklichkeit zum semiotischen Effekt einer Deutung, die noch dem Sinnwidrigsten, dem Bedeutungslosesten und Unverständlichsten einen verborgenen Sinn unterstellt.

Welche Konsequenzen aber hätte diese Form des Beobachtens – dieser Imperativ, nicht der naheliegendsten oder der institutionell abgesicherten Erklärung zu folgen, sondern mit peinlicher Genauigkeit alles Nebensächliche und alle Lücken zu beachten und fragwürdige Zeichen mit poetischem Geschick in einen verständigen Zusammenhang zu bringen – für die Betrachtung eines konkreten Fallbeispiels? Schauen wir uns dafür jenes denkwürdige Fallbeispiel an, mit dem Locards Einführung in die „wissenschaftlichen Grundlagen des Tatsachenbeweises“ beginnt. Wie zu sehen sein wird, dient dieses Beispiel, das der praktischen Einführung in Locards Methoden vorangestellt ist, nicht nur dazu, Locards Leser:innen von der Unerlässlichkeit der peinlich genauen Beobachtung sowie der „Annahme ohne vorherige Begründung“ zu überzeugen, sondern sie regelrecht performativ in die Kunst eines solchen Beobachtens und Vermutens einzuüben.

„Man erinnert sich der Umstände“, rekapituliert Locard: „[E]in Mädchen aus einer der besten Familien in Clermont‐Ferrand stirbt unter tragischen Umständen; Bruder und Mutter behaupten, sie hätten sie tot gefunden, erschlagen von den Trümmern der Zimmerdecke, die auf ihr Bett gefallen seien, dessen Vorhänge angesengt waren.“^54^ Die von einem Kollegen Locards durchgeführte kriminalistische Untersuchung, die nicht gründlich genug unternommen wurde, um zu einer Verurteilung zu führen, gibt Locard wie folgt wieder:

Die Beobachtungen am Tatort werden sehr mangelhaft vorgenommen, trotzdem die zunächst geglaubte Erklärung [der Mutter und des Bruders des getöteten Mädchens, A.S.] starke Unwahrscheinlichkeiten enthielt. Gleichwohl stellt man fest, daß der Stuckverputz nur mit Hilfe eines Stockes oder Besenstils heruntergeschlagen sein kann, und daß auch die von Trümmern verursachten Wunden den Tod nicht herbeigeführt haben können. Da das Opfer unverkennbare Spuren einer Vergewaltigung aufwies, kam man auf den Gedanken, daß es sich um ein Sittlichkeitsverbrechen mit tödlichem Ausgang und um den Versuch einer Brandstiftung handle. Da man feststellen will, daß das Verbrechen nicht von einem der Kläger begangen wurde, findet man schließlich ein blutiges Taschentuch, dass der unbekannte Täter unter dem Bett zurückgelassen hat, Einsteigespuren, ja sogar Fingerabdrücke außen am Fenster. Ein Ermittlungsrichter löst den anderen ab: sobald er sich mit dem Inhalt der Akten bekannt gemacht hat, wird er auch schon wie selbstverständlich abgelöst. Schließlich erhebt man gegen den Bruder Anklage wegen Vergewaltigung und Ermordung seiner Schwester. Ein Sachverständiger erklärte jedoch, daß er nicht zeugungsfähig sei. Vor dem Schwurgericht erweist sich die Anklage als unhaltbar und bricht zusammen. Das Verbrechen blieb ungesühnt.^55^

Der protokollarische Ton, mit dem Locard die Progression der missglückten Kriminaluntersuchung rekonstruiert, entspricht dem wenig außergewöhnlichen Gegenstand, der hier dargestellt wird: Locard beschreibt eine oberflächliche Spurensicherung nach alter Schule, die, gerade weil sie nur nachlässig durchgeführt wird und ergebnislos bleibt, recht konventionell dem Klischee der französischen Polizeiarbeit Anfang des 20. Jahrhunderts entspricht – einem Land, dessen Großstädte gebeutelt sind von einem Anstieg unaufgeklärter Gewaltverbrechen. Locards Darstellung der mangelhaften Beobachtungen am Tatort zielt deutlich darauf ab, ein Negativbeispiel zu statuieren, vor dessen Folie er die Überlegenheit der modernen Spurensicherung veranschaulichen kann. Bei aller Kritik scheint er dieser Tatortanalyse nach alter Gewohnheit aber immerhin zuzugestehen, „unverkennbare Spuren“^56^ und Tatwerkzeuge zu sichern, die wiederum zu nachvollziehbaren Schlüssen über den Tathergang führen (so etwa der Schluss vom Stuckverputz auf den vorsätzlich herbeigeführten Deckeneinbruch durch einen Besenstiel; oder von Einsteigespuren am Fenster auf einen Einbruch). Und bringt die Spurensicherung auch letztlich keine Beweise zu Tage, die vor Gericht zu einer Verurteilung führen, so zeugt die Tatsache, dass die Gerichtverhandlung mit einer Unschuldsvermutung ihren vorläufigen Abschluss findet, zumindest von der Fortschrittlichkeit eines modernen Rechtssystems, das *in dubio pro reo* entscheidet.

Bei genauerem Hinsehen wird indes ersichtlich, dass Locards Darstellung einem radikalen Verdacht Rechnung trägt, der dem Mord an Christophine implizit ein zweites Kriminaldelikt an die Seite stellt, welches den Fall schließlich als machtpolitisches Verbrechen lesbar macht: der Verdacht der Korruption der ermittelnden Behörden. Dieses zweite Verbrechen aber – und dies ist die strukturelle Pointe Locards – wird mit keinem Wort erwähnt und ist nur dann erkennbar, wenn man zwischen den Zeilen liest, scheinbare Nebensächlichkeiten notiert, die Details am Rande beachtet, die dem Ermittler entgingen, und sich darin versucht eine Hypothese zu entwickeln, die all diese verdächtigen Beobachtungen zueinander in Beziehung setzen und so erklärbar machen würde – kurz: wenn man eben jenes Verfahren anwendet, in das Locard im Kapitel zu den „wissenschaftlichen Grundlagen des Tatsachenbeweises“ einführen will, die Kunst des Beobachtens und die Kunst des Vermutens ohne vorherige Begründung.^57^

Folgt man Locard darin, im ersten Schritt der Kriminaluntersuchung lückenlos *alles* zu registrieren, und also auch diejenigen Details zu verzeichnen, die scheinbar bedeutungslos für das betrachtete Geschehen erscheinen, so fallen in dem zitierten Fallbeispiel eine Reihe von Erwähnungen auf, die auf den ersten Blick insofern strukturell zweitrangig erscheinen, als sie keinerlei Konsequenzen für die Progression der dargestellten Spurensicherung zu haben scheinen und von Locard konsequent unkommentiert bleiben. Bei näherer Betrachtung sind es indes gerade diese scheinbar nebensächlichen Details, die Fragen aufwerfen – und zwar nicht *obwohl*, sondern gerade *weil* ihnen weder vom Ermittler noch von Locard nachgegangen wird.

Bereits in den ersten Zeilen des Fallbeispiels führt Locard in diesem Sinne eine Reihe fragwürdiger Sachverhalte und zweifelhafter Entscheidungen an, die die Untersuchung des Tatortes strukturieren. Zunächst stellt er fest, dass erstens die Zeugenaussagen *trotz* starker Unwahrscheinlichkeiten zunächst geglaubt werden; und dass zweitens die Beobachtungen am Tatort mangelhaft vorgenommen werden. Ob dies absichtlich geschieht, oder ob hier grobe Fahrlässigkeit vorliegt, lässt Locard zwar zunächst dahingestellt; in jedem Falle erwecken diese Feststellungen seitens der Leser:innen aber das Bedürfnis nach einer Begründung, das, insofern diesem nicht nachgegangen wird, eine unbefriedigende Leerstelle hinterlässt, die Raum für Mutmaßungen gibt. Vielleicht noch merkwürdiger erscheint vor diesem Hintergrund zudem all jenes, was in der dargestellten Kriminaluntersuchung dezidiert ausgelassen wird, was dem „geschulten Sucher entgeht“: Verdächtige Tatsachen werden konsequent vom Ermittler ignoriert; die naheliegendsten Annahmen und logischen Reaktionen auf beobachtete Sachverhalte bleiben aus. Der Ermittler begnügt sich etwa damit festzustellen, dass es sich bei dem Einbruch der Decke weder um ein zufälliges Ereignis noch um die Todesursache handelt. Dass es aber eine verdächtige Korrelation zwischen dem fingiertem Deckeneinbruch und den Zeugenaussagen des Bruders und der Mutter der Ermordeten gibt, die sich für eben diesen Deckeneinbruch verbürgen, entgeht dem Kommissar. Auch die Möglichkeit, dass, wenn es sich schon bei der eingebrochenen Decke um ein Täuschungsmanöver handelt, das vom eigentlichen Verbrechen ablenken soll, auch der Brand fingiert sein könnte, wird vom ermittelnden Beamten nicht in Erwägung gezogen. Stattdessen werden die ungeprüften Brandspuren von ihm dazu benutzt, die „unverkennbare Vergewaltigung“ Christophines mit einem zweiten Verbrechen zu amalgamieren, welches den Verdacht von den Angehörigen des getöteten Mädchens ablenkt. So diagnostiziert der Beamte, „dass es sich um ein Sittlichkeitsverbrechen mit tödlichem Ausgang *und* um den Versuch einer Brandstiftung handle“, wobei Mutter und Bruder als Täter:innen kategorisch ausgeschlossen werden. Ob der ermittelnde Kommissar so sehr von der familiären Unschuld überzeugt ist, dass er die Möglichkeit ihrer Delinquenz gar nicht erst in Betracht zieht, oder ob es sich eben nicht um grobe Fahrlässigkeit, sondern die politisch motivierte Absicht handelt, „eine der besten Familien der Stadt“ nicht in Verruf zu bringen, lässt Locard zwar auch hier zunächst noch offen, doch die Kausalität, die er dem Kommissar unterstellt, müsste einem Leser, der mit Locards Methode der Beobachtung vertraut ist, doch mindestens fragwürdig vorkommen. Denn die Schlussfolgerung, dass es sich um einen Einbruch handelt – und also um die Misshandlung des Kindes durch eine unbekannte Person, die von außerhalb eindringt –, folgt nicht eben aus der Beobachtung einer eindeutigen Spur, die auf diesen Sachverhalt schließen ließe, sondern es verhält sich genau umgekehrt: Die scheinbaren Spuren des Verbrechens sind nach Locards Darstellung das Produkt eines Willens, der die Beobachtung motiviert und Spuren zu generieren versucht, nur um die bereits zuvor gefasste Schlussfolgerung der familiären Unschuld zu verifizieren: „*Da man feststellen* *will*, dass das Verbrechen nicht von einem der Kläger begangen wurde, *findet man* schließlich ein blutiges Taschentuch, dass der unbekannte Täter unter dem Bett zurückgelassen hat, Einsteigespuren, ja sogar Fingerabdrücke außen am Fenster.“^58^

Trotz aller Bemühungen des ermittelnden Beamten wird dann während des Gerichtsprozesses einer der Kläger, nämlich der Bruder, angezeigt, wie der letzte Absatz vermerkt. In dieser Passage werden implizite Hinweise auf eine Befangenheit der ermittelnden Behörden schließlich von der Exekutive auf die Judikative ausgeweitet. Es sind hier abermals die kleinen sprachlichen Mittel, die Locards Leser:innen vor die Aufgabe stellen, zwischen Fahrlässigkeit und Vorsatz der missglückten Kriminaluntersuchung zu unterscheiden: „Wie selbstverständlich“ werden Ermittlungsrichter abgelöst – und zwar nicht *obwohl*, sondern *sobald* sie mit dem Fall vertraut sind. Oder anders formuliert: Dass Richter ausgetauscht werden, ist eben nicht selbstverständlich, und dass diese ungewöhnliche Maßnahme sich nicht einmalig und zu einem zufälligen Zeitpunkt ereignet, sondern wiederholt immer genau dann, sobald die Beweisaufnahme einsetzen würde und Zeug:innen, Sachverständige und Tatverdächtige öffentlichkeitswirksam und eidesstattlich vernommen würden, nährt den Verdacht eines kalkulierten Eingreifens einer machtpolitischen Instanz, die ihre Verfügungsmacht über das Verfahren geltend macht.

So also landen wir ganz am Ende – und dies nur auf Basis einer gleichermaßen pedantischen wie spekulativen Auswertung sekundärer Quellen und ohne jemals eigene physische Beobachtungen angestellt zu haben – schließlich bei einem ganz anderen Verbrechen als der Ermordung Christophines. Ein Verbrechen, das Locard niemals direkt anspricht und das nur durch die Unterstellung einer Kausalität zwischen verdächtigen Einzelheiten gesehen werden kann, die sich an den Rändern des Fokussierten abzeichnen. Gelingt es Locard, dass seine impliziten Leser:innen im Fall Christophine schließlich nicht nur das Beispiel einer misslungenen Spurenuntersuchung, sondern neben diesem offensichtlichen noch einen zweiten, in ihm verborgenem Sachverhalt erkennen, so führt er performativ vor, wie sich Sinnbildung im Zeichen des Verdachts vollzieht, sobald man jedem Detail einen verborgenen Sinn unterstellt. Der Korruptionsverdacht ist das manifeste Produkt eines analytischen Paradigmas, das im buchstäblichen Sinne des Wortes [παρά + δείκνυμι]^59^ *nebenbei* begreiflich macht, was an der Oberfläche nicht zu sehen ist.

Wären spätestens hier, indem Beobachtungen zu einer konkreten Mutmaßung verdichtet wurden, die wichtigsten Schritte seiner Kriminaluntersuchung abgeschlossen, so bringt Locard seine Aufzeichnungen des Fallbeispiels mit einer belohnenden Geste an seine Leser:innen zu einem Ende. „Was hätte hier eine Beobachtung ergeben, die nach den Vorschriften der Technik durchgeführt worden wäre?“, fragt er seine Leser:innen rhetorisch. Und seine Beantwortung dieser Frage zeigt, wie konsequent Locards Glaube an den Erfolg der Vermutung ohne vorherige Begründung die Dramaturgie seiner Fallbeispiele bestimmt. Er kontrastiert die misslungene Spurensicherung seines Kollegen mit einer hypothetischen Untersuchung nach seiner eigenen Methode, unterstützt durch die Mittel der Technik. Diese aber, so Locards Pointe, leistet nicht mehr und nicht weniger als die Validierung all jener Verdachtsmomente, die durch eine „peinlich genaue Beobachtung“ der Details im Text ohnehin schon verzeichnet wurden: Eine Analyse der Brandspuren hätte ergeben, dass es sich beim Brand, „nicht um einen Unglücksfall, sondern nur um die Vortäuschung eines solchen handelte.“^60^ Eine Untersuchung der Fensterabdrücke hätte ergeben, dass es sich bei dem vermeintlichen Einbruch um nichts als eine „*Romanhafte Erzählung* über Kletterspuren“^61^ handelt. Eine genaue Untersuchung der Blutflecke hätte das blutige Taschentuch, das der ermittelnde Kommissar sicherstellte, als „Erfindung (im eigentlichen Wortsinne)“^62^ entlarvt. Und damit wären schließlich sämtliche Spuren eines Einbruchs und einer Vergewaltigung, die bei der Spurensicherung gefunden wurden, als Fälschungen entlarvt, die von ausgerechnet derjenigen Person platziert wurden, die die Tat aufklären sollte. Die Tat selbst, der Mord an Christophine, bleibt indes weiterhin unaufgeklärt.

Tatsächlich ist es wohl einzig der Verdacht der Korruption – bzw. das bewusste Hervorrufen dieses Verdachts bei seinen Leser:innen –, welcher Locard überhaupt dazu veranlasst haben dürfte, den Fall Christophines als erstes Exempel seiner methodischen Einführung in „Die wissenschaftlichen Grundlagen des Tatsachenbeweises“ anzuführen. Denn Locard erscheint nicht besonders interessiert daran, den eigentlichen Tatbestand der Ermordung zu lösen – dieses, wie Locard schreibt, „Rätsel […], das gewiss nicht uninteressant, aber auch nicht besonders in Dunkel gehüllt war.“^63^ Locards Interesse gilt nicht dem, was zu sehen ist, oder dem, was rein sachlich begründet werden kann, sondern im Gegenteil dem, was tatsächlich im Dunkeln verborgen ist, was nicht untersucht wurde und nicht zur Anklage kam – und dessen Existenz einer gezielten Befragung und spekulativen Entzifferung von Indizien bedarf, die ein Geheimnis unter der Oberfläche indizieren. Oder methodisch gewendet: Locard ist interessiert an der Frage, wie man zu einer logischen Schlussfolgerung über etwas kommen kann, was nur durch einen Verdacht gegen das Offensichtliche erkannt werden kann.

Die Ironie, die Locards „Lösung“ des Falles Christophine mit sich bringt, ist bestechend: Das effektivste Mittel, um den gefährlichen Anteil des „Romanhaften“, der „Erzählung“ und der „Erfindung“ aus der Kriminaluntersuchung zu verbannen, ist eine Methode, die im Kern nichts anderes als die Anwendung eben dieser erzählerischer Prinzipien auf den Kriminalfall vollzieht: der exzessive Gebrauch der Einbildungskraft, das buchstäbliche Erfinden von kausalen Zusammenhängen zwischen verdächtigen Zeichen, das fabulatorische Auserzählen vermeintlicher Lücken und schließlich das Vermögen, die Gesamtheit unbewegter Zeichen in die Sukzession eines Handlungsverlaufs zu bringen, der sämtliche dieser Operationen nachvollziehbar macht –kurz gesagt: das Erzählen einer Geschichte.

## Footnotes

1
Locard 1920, S. 218.

2
Zum Fall Fabry‐Rollin siehe ebd., S. 95 und 106. Zu Locards Faszination mit A.E. Poe siehe ebd., S. 190.

3 Zum Begriff der „unwiderlegbaren Vermutungen“ siehe ebd., S. 18. Dort heißt es: „Es gibt Vermutungen, über die man streiten kann. Einige, die im Strafgesetz festgelegt sind, stellen wirklich gesetzlich zulässige Beweise dar: so wird jeder Landstreicher, der mehr als 100 Francs bei sich trägt, nach Artikel 273 StrGB bestraft, ohne dass er den Beweis erbringen darf, daß er dieses Geld auf ehrliche Weise erworben hat. Ebenso wird der Soldat, der zur Flucht vor dem Feinde aufwiegelt […] bestraft, ohne daß der Beweis zugelassen wird, daß er nicht mit dem Feinde im Einverständnis gewesen sei. Im ersteren Fall liegt die unumstößliche Vermutung des Diebstahls, im zweiten die des Kriegsverrats vor.“ Siehe auch ebd., S. 15.

4 Dazu ebd., S. 95.

5 Zum Begriff der „Wissenschaft des Verdachts“ siehe Vogl 1991.

6 „Die Wissenschaftlichen Grundlagen des Tatsachenbeweises“ lautet der Titel des letzten Kapitels der *Kriminaluntersuchung*. Siehe Locard 1920.

7 Im Folgenden soll in erster Linie jene spekulative Praktik beschrieben werden, die Locard als „Verfahren der Annahme ohne vorherige Begründung“ bezeichnet. Weitere spekulative Praktiken, die in der *Kriminaluntersuchung* vorgestellt werden, umfassen den sogenannten „Ähnlichkeitsschluss“ sowie das „Hineinversetzen in die tatverdächtige Person“. Ebd., S. 184–219.

8 Ebd., S. 43.

9 Ebd., S. 65.

10 Locards kategorielle Abwendung von Zeug:innenaussagen und Geständnissen trägt dabei nicht nur einem berechtigten Zweifel an der Glaubwürdigkeit gewisser Zeug:innen oder der Aussagekraft von unter polizeilichem Druck erzwungenen Geständnissen Rechnung, sondern auch den sexistischen Vorurteilen, die den Beginn dieser kriminologischen Disziplin kennzeichnen: So erklärt Locard, dass es „eine Reihe von Zeugen [gäbe], die man ohne Widersinn als zeitweilig geistesgestört bezeichnen kann: die Frau und das Kranke syphilitische Kind. Während des monatlichen Wechsels, in der Schwangerschaft, in der ersten Zeit nach der Entbindung, beim Ausbleiben der Periode ist die Frau eine verdächtige Zeugin, da sie voreingenommen und nicht voll verantwortlich ist. Sie ist dann fabelsüchtig.“ Ebd., S. 62.

11 Ebd., S. 184. Das Zitat entnimmt Locard den Schriften Francis Bacons.

12 Ebd., S. 22.

13 Das 1823 von Purkynê, dem Begründer der Histologie, erstmals vorgestellte und 1888 von Galton populär gemachte Verfahren der Daktyloskopie wurde zwar bereits in den 1890 Jahren zur Personenidentifikation eingeführt, bis zu Locards Formalisierung dieses Verfahrens wurden aber noch keine Verurteilungen auf Basis daktyloskopischer Gutachten ausgesprochen.

14 Locards Werk adaptiert, bündelt und formalisiert eine Reihe von Methoden, die, ursprünglich im Bereich der Medizin und der Anthropologie entwickelt, in der zweiten Hälfte des 19. Jahrhunderts von Rodolphe Archibald Reiss, Alexandre Lacassagne, Cesare Lombroso und Alphonse Bertillon graduell bereits im Feld der Kriminalistik erprobt wurden. Siehe Reiss 1911.^⁠^

15 Ebd., S. 195.

16
Ginzburg 1985, S. 125.

17 Locard 1920, S. 190.

18 Zur kriminalhistorischen Einordnung der „Verdächtigungsstrategien“ in der Kriminalistik und Kriminologie um 1900 siehe Bachhiesl 2021. Zum Einfluss der Literatur auf Locards Werk siehe Chevallier 2020.

19 Zum Begriff der Sattelzeit siehe Koselleck 1972, S. 10–28.

20 Zum epischen Muster des *suspense* im Detektivroman siehe Vogl 2010, S. 71.

21 Locard 1920, S. 186.

22 Ebd., S. 187.

23 Ebd., S. 128.

24 Ebd., S. 187. Hervorhebung des Autors.

25 Ebd.

26 Ebd., S. 156.

27 Ebd., S. 187.

28
Ginzburg 1985, S. 125.

29 Ebd., S. 136.

30 Ebd., S. 134.

31 Ebd., S. 163.

32 Ebd.

33 Locard 1920, S. 190.

34 Ebd., S. 187.

35 Ebd., S. 190.

36 Ebd., S. 188. Hervorhebung des Autors.

37 Der Begriff des „lateralen Denkens“ wurde erst in den 1970ern, ein halbes Jahrhundert nach Locard, mit der Verbreitung der Schriften Edward de Bonos bekannt. Er eignet sich jedoch zur Beschreibung der Locard'schen Methode, da er die Abkehr von konventionellen Erklärungsmodellen zugunsten von intuitiven und assoziativen Modellen des Ratens bezeichnet. Dazu: Bono 1967.

38 Dazu: Schmidt 1985.

39 Locard 1920, S. 192.

40 Ebd., S. 190.

41 Ebd., S. 190. Hervorhebung des Autors.

42 Ebd., S. 192. Hervorhebung des Autors.

43 Ebd., S. 190.

44 Ebd.

45 Ebd.

46 Ebd.

47 Zit. nach Roth 1989, S. 30.

48 Locard 1920, S. 190.

49 Ebd.

50 Wie Locard stellt Sherlock Holmes diese Praktik in *A Study in Scarlet* als Begabung vor, Fälle lösen zu können, ohne selbst genauere Beobachtungen angestellt zu haben. Siehe Conan‐Doyle 1892, S. 30.

51
Peirce 1935–1966.

52 Zum Vergleich von Raten und Abduktion siehe Vogl 2010, S. 75.

53 Ebd., S. 76.

54 Locard 1920, S. 186.

55 Ebd.

56 Ebd.

57 Siehe ebd., S. 185–190.

58 Ebd., S. 186. Hervorhebung des Autors.

59 Paradigma von παρά (pará): neben + δείκνυμι (deíknymi): zeigen, begreiflich machen.

60 Ebd.

61 Ebd. Hervorhebung des Autors.

62 Ebd.

63 Ebd.
